# Fat perception in the human frontal operculum, insular and somatosensory cortex

**DOI:** 10.1038/s41598-018-30366-0

**Published:** 2018-08-07

**Authors:** Thomas Wistehube, Michael Rullmann, Claudia Wiacek, Peggy Braun, Burkhard Pleger

**Affiliations:** 1 Department of Neurology, Max Planck Institute for Human Cognitive and Brain Sciences, Stephanstr. 1a, 04103 Leipzig, Germany; 2 Collaborative Research Centre 1052 Obesity Mechanisms, University of Leipzig, Leipzig, Germany; 3 IFB AdiposityDiseases, Leipzig University Medical Centre, Liebigstr. 20, 04103 Leipzig, Germany; 4 Department of Nuclear Medicine, University Hospital Leipzig, Liebigstr. 18, 04103 Leipzig, Germany; 5 Institute of Food Hygiene, Center of Veterinary Public Health, University of Leipzig, An den Tierkliniken 1, 04103 Leipzig, Germany; 6 BMBF nutriCARD, Center of Veterinary Public Health, University of Leipzig, An den Tierkliniken 1, 04103 Leipzig, Germany; 7 Department of Neurology, BG University Hospital Bergmannsheil, Ruhr-University Bochum, Bürkle-de-la-Camp Place 1, 44789 Bochum, Germany; 8 Collaborative Research Centre 874 Integration and Representation of Sensory Processes, Ruhr-University Bochum, Bochum, Germany

## Abstract

Here, we combined magnetic resonance imaging with lesion-symptom mapping in patients with chronic brain lesions to investigate brain representations of sugar and fat perception. Patients and healthy controls rated chocolate milkshakes that only differed in sugar or fat content. As compared to controls, patients showed an impaired fat, but not sugar perception. Impairments in fat perception overlapped with the anterior insula and frontal operculum, together assumed to underpin gustatory processing. We also identified the mid-dorsal insula as well as the primary and secondary somatosensory cortex - regions previously assumed to integrate oral-sensory inputs. These findings suggest that fat perception involves a specific set of brain regions that were previously reported to underpin gustatory processing and oral-sensory integration processes.

## Introduction

During eating, exteroceptive food-related sensory signals from taste and olfactory receptor cells seem to activate the insular cortex together with the frontal operculum which together are assumed to underpin gustatory processing^1–4^. Studies using direct stimulation of the insular cortex as performed in the context of pre-surgical invasive exploration in epilepsy patients support this assumption. Penfield and Faulk were the first to report gustatory sensations evoked by direct stimulation of the lower part of the insular cortex in those patients^5^. Their intraoperative explorations were mostly performed after removal of the temporal operculum, left largely unexplored the upper and median part of the insular cortex. Mazzola *et al*. recently filled this gap^6^. Their findings suggest a spatial overlap between gustatory, olfactory, and oral somatosensory representations in the mid-dorsal part of the insular cortex. Together, these findings agree with meta-analysis of neuroimaging data^4,7,8^. As compared to primates, the insular “taste” cortex in humans was found further caudally suggesting that its location may have “migrated” during evolution^8–10^.

The insular cortex in the human brain lies deep within the lateral sulcus, almost surrounded by the groove of the circular sulcus and covered by the insular opercula. Not only the insular cortex itself, but also its frontal and parietal opercula were shown to underpin gustatory processes. In agreement with functional brain imaging studies^4,7,8^, Hausser-Hauw and Bancaud found gustatory sensations by direct stimulation of both regions^11^. The parietal operculum is the location of the secondary somatosensory cortex that together with the primary somatosensory cortex on the postcentral gyrus was shown to respond to fat emulsions in the mouth, supporting the assumption that somatosensory regions play a role in fat perception^12,13^.

In the present study, we investigated sugar and fat perception in patients with chronic brain lesions. We included sugar tests to assess brain representations of a primary taste quality, namely sweetness. Fat tests were included to identify brain representations coding the textual property of food. Sugar and fat ratings of patients were compared to healthy participants to identify sugar and fat categories that differed significantly between patients and controls. To assess brain areas involved in an impaired sugar or fat perception in those categories, we next used magnetic resonance imaging (MRI) data together with voxel-based lesion-symptom mapping (VLSM 2.55)^14^. We hypothesized impairments in sugar perception that overlap with lesions in the anterior insular cortex and frontal operculum. For fat perception, we assumed an association with the mid-dorsal insula and the somatosensory cortex, assumed to integrate gustatory, olfactory, and oral somatosensory representations required to identify the food’s contextual property.

## Results

### Sugar and fat perception

Twenty-five patients (fifteen men; average age = 50.04 ± 10.4 (standard deviation) years; average body mass index (BMI) = 25.5 ± 3.2 m^2^/kg (height^2^/weight)) with chronic brain lesions (see Table 1 for clinical data and Supplementary Figure 1 for individual lesion maps), as well as twenty-five healthy matched control participants (fifteen men; average age = 50.32 ± 11.2 years; average BMI = 24.8 ± 2.8 m^2^/kg) rated chocolate milkshakes with an increasing amount of fat (f1 to f3) and sugar (s1 to s3).

**Table 1:** Patients’ clinical data.

Patient	Age (years)	Gender (F = female, M = male)	Body Mass Index (BMI) (m^2^/kg)	Time from onset to study (months)	Origin of lesion
P1	53	F	29.4	203	left middle cerebral artery (MCA) stroke
P2	50	M	27.4	100	left MCA stroke
P3	27	F	21.8	66	traumatic subarachnoid hemorrhage (SAH)
P4	61	F	24.3	67	SAH due to aneurysma of right MCA
P5	51	M	23.6	60	left MCA stroke
P6	58	M	24.5	71	SAH due to aneurysma of right MCA
P7	59	F	21	41	right MCA stroke
P8	42	F	21.4	32	traumatic SAH
P9	54	F	24.3	32	SAH due to aneurysma of anterior communicating artery
P10	53	F	21	22	left MCA stroke
P11	67	F	29.6	29	left MCA stroke
P12	57	F	25.6	23	SAH due to aneurysma of right MCA
P13	28	M	27.5	20	traumatic brain injury (TBI)
P14	41	M	24.7	19	TBI
P15	53	M	25.1	15	right MCA stroke
P16	35	M	32.7	17	right MCA stroke
P17	55	M	29.4	12	TBI
P18	35	M	24.1	13	TBI
P19	52	M	29.9	15	SAH due to aneurysma of anterior communicating artery
P20	60	M	27	10	right MCA stroke
P21	62	F	26	11	SAH due to aneurysma of anterior communicating artery
P22	43	M	24.4	79	SAH due to aneurysma of anterior communicating artery
P23	47	M	21	51	left MCA stroke
P24	55	M	23.1	49	right MCA stroke
P25	53	M	28.7	10	right MCA stroke

The table lists patients’ age, gender, BMI, the time from onset to study as well as the origin of lesion. For the corresponding brain lesion pattern of each patient please refer to Supplementary Figure 1.

**Figure 1:**
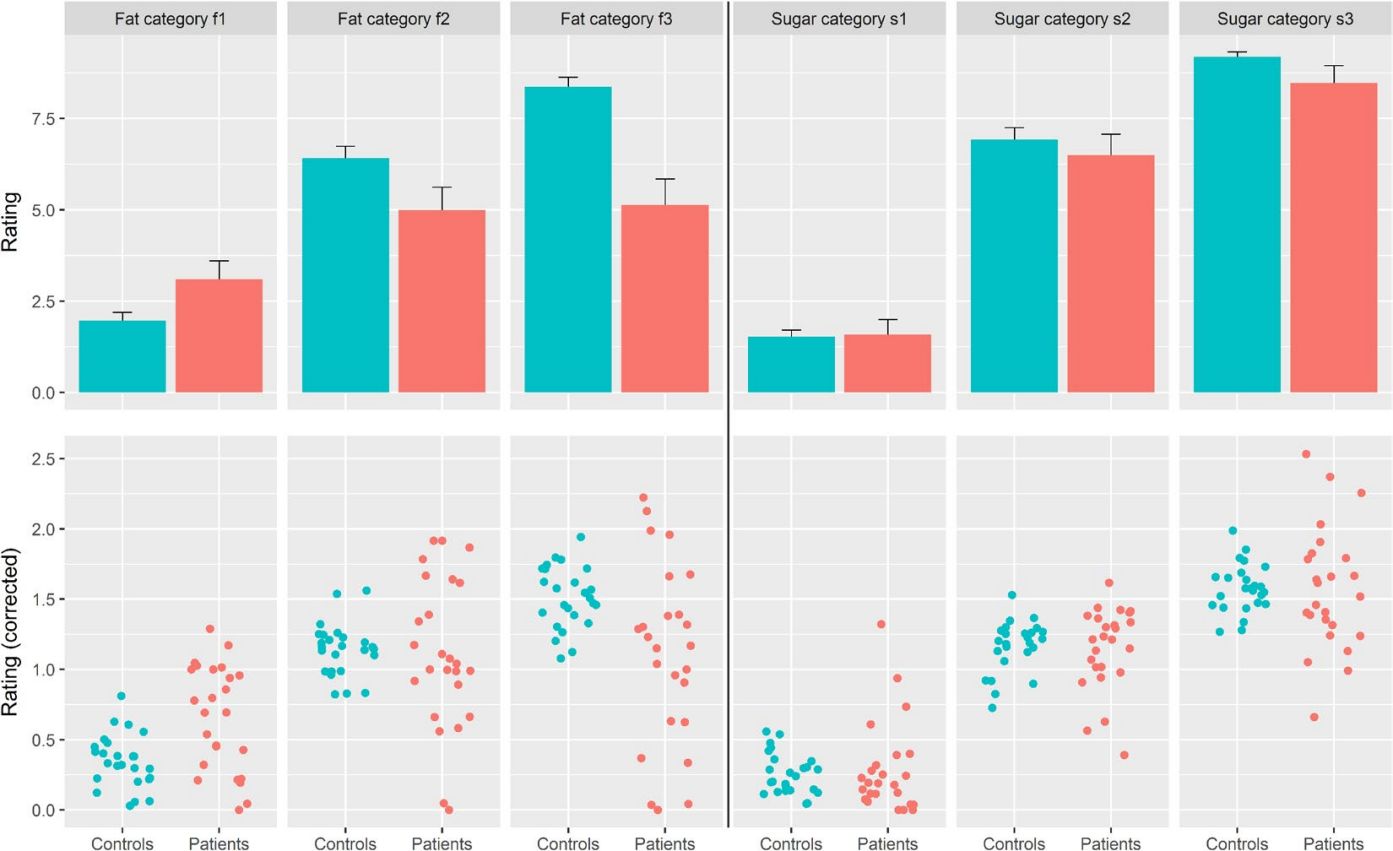
Sugar and fat ratings. Shown are patients’ and controls’ sugar and fat ratings for the three chocolate milkshakes containing increasing amounts of either sugar (s1 to s3) or fat (f1 to f3). (**a**) The bar plots represent the mean, the whiskers the standard error of the absolute ratings of patients (red) and controls (green) (**b**) The scatter plots show the mean-corrected ratings of each participant. Patients are colored in red, controls in green. We found no significant differences in sugar ratings between patients and controls. For fat ratings, we found significant differences for f1 and f3, but not for f2.

Contrarily to our a-priori hypotheses (see Introduction), we found no significant differences for the three sugar categories between patients and controls (s1: p = 0.76; s2: p = 0.84; s3: p = 0.93; see Fig. 1a for absolute values, and 1b for mean-corrected values).

For the three fat categories, we found significant differences for f1 (p = 0.00072) and f3 (p = 0.005), but not for f2 (p = 0.81) (Fig. 1a,b). Both significant t-tests were corroborated by two-sample permutation test using Welch’s t (B = 10000) (f1: t-statistic = −3.61, p = 0.0016; f3: t = 2.93, p = 0.005). While for f1 we found that patients overrated the fat amount, for f3 they underrated it (f1: patients mean 0.65+/− 0.08 (standard error), controls 0.35+/− 0.04; f3: patients 1.11+/− 0.13, controls 1.51+/− 0.04). All patients were able to detect fat per se.

For those ratings of patients that proved significant differences (i.e., f1 and f3), we applied VLSM.

### Changes in taste, flavoring and eating pleasure after brain injury/hunger, thirst and degree of tiredness

Patients who overrated f1 were more often told to use more spice, sugar or salt, whereas patients who underrated f3 were told to use less spice, sugar or salt. For the questions whether patients were eating more, less or the same amount of food after brain injury, we found no differences between patients who perceived f1 and f3 as normal as compared to those who presented a perceptual deficit in those fat categories. For f1, we found that 40% of patients who perceived f1 as normal nevertheless reported changes in food preferences. Forty-seven % of the same patients also reported that eating became boring. For those who overrated f1, only 20% reported changes in food preferences and only 10% reported that eating became boring. For f3, we found that 27% of patients who perceived f3 as normal reported changes in food preferences. Sixty-four % of the same patients also reported that eating became boring. For those who underrated f3, 36% reported changes in food preferences and 29% reported that eating became boring. These findings suggest a relative unawareness for deficits in fat perception (Table 2).

**Table 2:** Changes in taste, flavoring and eating pleasure after brain injury/hunger, thirst, and degree of tiredness.

Question	Fat 1 – deficit	Fat 1 - normal	Fat 3 – deficit	Fat 3 - normal
Changes in taste perception? (yes/no)	10% yes	53% yes	29% yes	45% yes
Too much spice? (yes/no)	40% yes	27% yes	7% yes	64% yes
Too much salt? (yes/no)	30% yes	27% yes	14% yes	45% yes
Too much sugar? (yes/no)	40% yes	7% yes	7% yes	64% yes
Changes in food preferences? (yes/no)	20% yes	40% yes	36% yes	27% yes
Did eating become boring? (yes/no)	10% yes	47% yes	29% yes	64% yes
Eating more? (yes/no)	10% yes	13% yes	7% yes	9% yes
Eating less? (yes/no)	30% yes	34% yes	36% yes	27% yes
Eating the same amount? (yes/no)	60% yes	53% yes	57% yes	64% yes
VAS hunger (1–10)	2.06 + /− 0.18	1.69 + /− 0.16	1.7 + /− 0.11	2.05 + /− 0.24
t-test hunger, deficit vs. normal	p = 0.67		p = 0.68	
VAS thirst (1–10)	4.08 + /− 0.29	2.3 + /− 0.17	2.04 + /− 0.17	4.4 + /− 0.26
t-test thirst, deficit vs. normal	p = 0.12		p = 0.03	
VAS “how well did you sleep last night” (1–10)	6.77 + /− 0.29	5.4 + /− 0.2	6.26 + /− 0.24	5.69 + /− 0.23
t-test sleep, deficit vs. normal	p = 0.27		p = 0.65	

For the latter three measures we applied visual analogue scales (VAS) ranging from 0 to 10. VAS ratings between patients with a deficit in taste perception and those not presenting such a deficit were applied to unpaired t-tests.

With visual analogue scales we assessed hunger, thirst and the degree of tiredness before the experiments. We found no differences between patients for hunger or the degree of tiredness. For thirst, we found that patients who perceived f3 as normal were thirstier before the experiments than those who underrated f3. To assess whether thirst was generally associated with the perception of f3, we additionally computed Pearson’s correlation analysis of both ratings across all 25 patients. We found no association between both ratings (r = 0.28, p = 0.176), suggesting that thirst was not associated with impairments in the perception of the highest fat content (Table 2).

### MRI-based voxel-by-voxel lesion-symptom mapping

**Figure 2:**
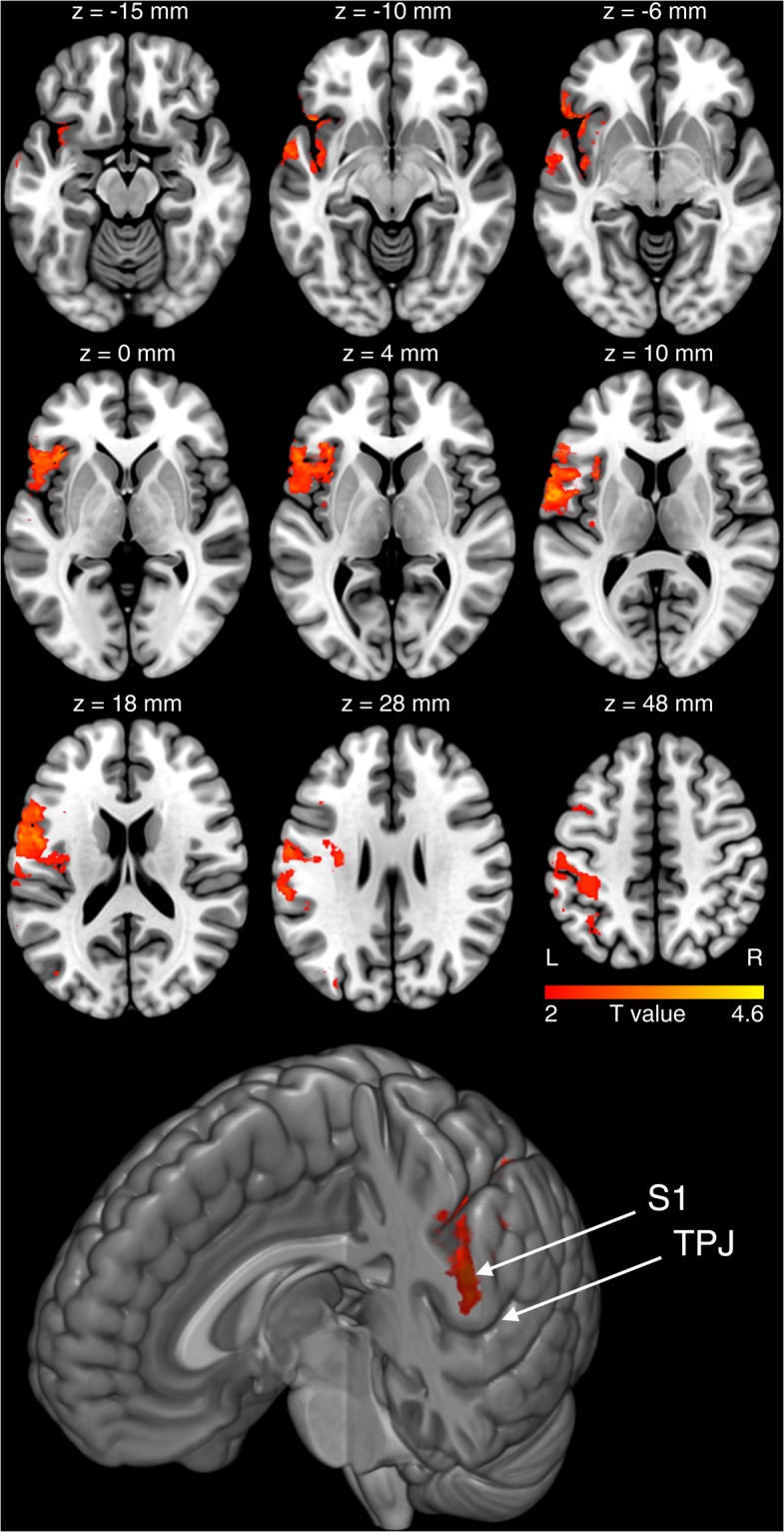
MRI-based lesion symptom mapping. Shown are the brain regions that overlap with an impaired fat perception in the category f3. For f1, we also found significant differences in fat ratings between patients and controls. VSLM, however, revealed no significant effects for f1 (see Fig. 1 for corresponding sugar and fat ratings). The ‘z’ below each brain slice indicates the corresponding z-coordinate of that slice in MNI space. ‘L’ indicates the left and ‘R’ the right hemisphere. We found that an impaired fat perception in f3 overlapped with the anterior insular cortex together with the frontal operculum. Both regions are together assumed to underpin gustatory processing. We also found the mid-dorsal insula together with the primary and secondary somatosensory cortex - regions that are assumed to integrate sensory-oral inputs. At the bottom of the figure we show a render brain with a coronal cut through the primary somatosensory cortex. TPJ indicates the temporoparietal junction, S1 the primary somatosensory cortex. The effect in f3 appears to overlap with the representations of the face including the oral cavity as well as the tongue.

For f1, the t-statistics revealed no significant cluster even when applying a non-significant threshold at p = 0.05. For f3, we identified the anterior insular cortex (superior cluster: x = −40, y = 14, z = 16, T = 2.18; x = −32, y = 16, z = 14, T = 3; inferior cluster: x = −62, y = 6, z = 6, T = 4.51; Fig. 2), the frontal operculum (x = −40, y = 28, z = 14, T = 2.11; x = −42, y = 18, z = 8, T = 2.38; x = −52, y = 34, z = 0, T = 2.58), the mid-dorsal insular cortex (x = −40, y = 0, z = 12, T = 2.28; x = −36, y = 0, z = 8, T = 2.28; x = −36, y = 6, z = −6, T = 2.46), the primary (x = −48, y = −10, z = 32, T = 2.31) as well as the secondary somatosensory cortex (x = −40, y = −18, z = 4, T = 2.32).

At the bottom of Fig. 2 we show a render brain with a coronal cut through the primary somatosensory cortex. The effect in f3 appears to overlap with the representations of the face, including the oral cavity, as well as the tongue.

To assess differences between both brain hemispheres, we additionally analyzed the data of patients with left-sided and right-sided lesions separately. Unthresholded t-maps of both sides resembled the analysis of the flipped data (Supplementary Fig. 2).

To test whether overrating f1 and underrating f3 was associated with lesions in the same set of brain areas, we first questioned whether the same patients who overrated f1 also underrated f3. Out of the fourteen patients who underrated f3, only five also overrated f1. This suggests partly different symptom-lesion associations. Next, we computed lesion overlay images of patients who overrated f1 and of those who underrated f3 (Supplementary Fig. 3). The comparison between both overlay images must be handled carefully since we could not apply the data to a statistical analysis. Visual comparison nevertheless suggests that patients who underrated f3 additionally presented lesions in the frontal operculum, whereas patients who overrated f1 did not present lesions in this region. Whether the additional presence of lesions in the frontal operculum is associated with differences in fat perception should be investigated in future studies that have the power to answer this question statistically and not just descriptively.

## Discussion

In the present study, we show that an impaired fat perception overlapped with lesions in the anterior insular cortex, frontal operculum, the mid-dorsal insula as well as the primary and secondary somatosensory cortex.

Fat in the mouth leads to enhanced activity not only in gustatory regions, such as the medial orbitofrontal cortex (OFC)^12^, the insular cortex^12,15,16^ or the frontal operculum^17^, but also in the anterior cingulate cortex^13^, assumed to support autonomic functions, reward anticipation, decision-making, and impulse control, in the hypothalamus^12,15,16^, as the brain’s homeostatic control site, in the amygdala^13,18^, assumed to integrate memory, learning, decision-making and emotional reactions, as well as the primary and secondary somatosensory cortex^13^ involved in somatosensory perception and recognition^19,20^. Together these brain imaging studies suggest a large neuronal network, including homeostatic (hypothalamus), somatosensory and reward areas (putamen, pallidum, amygdala) that together with gustatory regions (insular cortex, frontal operculum, medial OFC) work in concert while processing fat in the mouth^21^. The present findings suggest that a subset of those regions, namely the anterior insular cortex, frontal operculum, the mid-dorsal insula, as well as the primary and secondary somatosensory cortex, are involved in rating the food’s fat content emphasizing their role in basic processes underpinning fat perception. For sugar ratings, we found no differences between patients and controls. This could point to other regions, outside the present lesion pattern, that may be involved, such as the (dorsal-)medial OFC or prefrontal regions. An alternative interpretation is that the perception of sugar is faster compensated than the perception of fat, since sugar represents the brain’s main source of energy elementary for energy-consuming plastic brain processes also required for rehabilitation after brain damage. Besides a case report of a 75-year-old woman with a complete left middle cerebral artery infarct and taste deficits^22^, we could not find any other evidence supporting this hypothesis. Six months after stroke, the women complained of “distaste” for her preferred foods. This was most severe over the first several weeks to months following the stroke, resulting in her eating less and losing weight (~14 lbs or 6.35 kg). Nine months post stroke the patient had identified several foods that she could taste and enjoy. At the same time, sweet foods and sugar tasted as expected, and she was able to enjoy chocolate. These observations in a patient aware of “distaste” support our assumption that sugar perception may recover faster than other tastes. But it is only a case report. Studies in acute brain-lesioned patients and longitudinal studies using also other taste qualities are required to support this hypothesis.

Activity in the anterior insular cortex in response to fat ingestion is one of the best reproduced brain imaging effects^12,13,15^. Together with the frontal operculum its functional associations seem to range from basic processes underpinning taste perception^1–3^ to higher cognitive processes of gustatory sensations^23^. For a long time, it was believed that both regions are only active during eating or drinking. Exteroceptive sensory signals arising from the food’s taste and smell were assumed to activate olfactory receptor cells that propagate associated inputs through the thalamus to the frontal operculum and anterior insular cortex, where stimulus identity and intensity are merged into a stable representation, independent of the homeostatic or motivational state^1–3,24^. This assumption was challenged by recent findings suggesting activity in both regions elicited by visually presented food, independent of signals from peripheral taste or olfactory receptor cells. This suggests that gustatory regions in not only mice^25^ but also in humans^23^ contribute to the ability to imagine food and taste. Food and taste evaluation, imagery and their influence on food choices are an essential function for survival. Their implementation in the gustatory cortex may therefore represent a well-preserved evolutionary effect^26^.

Besides the anterior insula, we found that also the mid-dorsal insular cortex was associated with an impaired fat perception. This insular sub-region in the human brain was recently shown to contain spatially overlapping representations for gustatory, olfactory, and oral somatosensory sensations suggesting its role in the integration of oral-sensory inputs^6^, also required to perceive and rate the food’s fat content. The association between fat perception and gustatory regions adds to the controversial discussion whether fat belongs to the primary taste qualities, such as sweet, salty, sour, bitter and umami^21^.

Regarding the insula’s more general cognitive functions, studies suggest its involvement in processes related to the sense of ownership and agency^27^, or the subjective awareness and affective processing of bodily signals^28,29^. The anterior part of the insular cortex is specifically assumed to play a major role in viscerosensory^30^ and interoceptive processing^28,29^. The posterior insula is thought to contain perceptual representations for bodily awareness^31^. Projections between the anterior and posterior insula were thus assumed to relate to our subjective awareness of our body and bodily emotions^32^.

The parietal operculum covers the insular cortex and contains the secondary somatosensory cortex that together with the primary somatosensory cortex and the insular cortex represents the ventral pathway of somatosensory processing involved in somatosensory perception and recognition^19,20^, also in the context of oral-somatosensory sensations^13,18,21^. In line with these interpretations, we found that the effect in the primary somatosensory cortex overlapped with the representations of the face, including the oral cavity, as well as the tongue (see the S1 effect projected on a render brain at the bottom of Fig. 2).

The frontal operculum has been suggested as a key node in a network for exerting control over cognitive processes. It seems to regulate the activity in relevant or irrelevant brain representations for response selection^33^, possibly also in the context of fat perception.

The present findings provide evidence for the joint involvement of the anterior/mid-dorsal insula, the frontal operculum as well as the primary and secondary somatosensory cortex in fat perception. However, our study does not allow to distinguish between impairments in taste, olfaction or somatosensory perception since participants in our study rated probes of milkshakes. Their consumption provokes effects in all three sensory domains and hence in corresponding brain representations. Future VLSM studies should apply more specific tests to separate taste, olfaction or somatosensory functions as well as their distinct representations in the human brain.

## Methods

### Patients

MRI scans of potentially suitable patients were collected from a database at the Max Planck Institute for Human Cognitive and Brain Sciences in Leipzig, Germany. The database contains more than 3800 MRI scans and examination reports of young brain lesion patients. The inclusion criteria were brain lesions in either the right, left or both brain hemispheres. Spatial neglect, serious communication problems, cognitive and language deficits, nut allergy, as well as diabetes and/or lipometabolic disorders were exclusion criteria. We identified 55 patients who met those criteria. We called all of them, but most patients declined our invitation because of reasons such as anxiety, too busy, total duration of experiments, or disinterest.

Ten patients (five women; average age = 47.1 ± 12.22 years) who agreed to participate presented brain lesions in the left hemisphere, ten patients (three women; average age = 54.6 ± 7.43 years) presented lesions in the right hemisphere and another five patients (two women; average age = 46.8 ± 10.43 years) presented lesions in both hemispheres (see Table 1 for patients’ age, gender, BMI, the time from onset to study as well as the origin of lesion, and Supplementary Figure 1 for the corresponding individual lesion maps).

All patients were tested in the chronic lesion phase (10 to 202 months post onset). The study was approved by the ethics committee of the University Clinic Leipzig and conducted according to the ethical guidelines of the Declaration of Helsinki.

Before the experiments, we used yes/no questions to assess changes in taste, flavoring and eating pleasure after brain injury. To this end, we asked questions, such as “Did you experience any changes in taste after brain injury?”, “Are you sometimes told that you are using too much spice/salt or sugar?” (i.e., three questions), “Did your food preferences change after brain injury (e.g., favorite dish)?”, “Do you sometimes experience eating as boring?”, and “Do you eat more/less/the same after brain injury?” (i.e., three questions). Furthermore, we asked patients to rate their hunger, thirst, and degree of tiredness on three visual analogue scales (VAS) ranging from 0 to 10. To this end, we asked “How hungry do you feel now?” ranging from “not hungry at all” to “very hungry”, “How thirsty do you fell now?” ranging from “not thirsty at all” to “very thirsty”, and “How well did you sleep last night?” ranging from “bad” to “good”. The line of each VAS was 100 mm long. The distance between 0 and the cross made by the patient in mm was applied to further analyses.

We acquired high-resolution whole-brain 3D standard T1-weighted anatomical images and T2-weighted fluid-attenuated inversion-recovery (FLAIR) images for each patient. In stroke patients, we used the FLAIR images in parallel to the high-resolution T1 images to better delineate the lesion borders. All images were acquired with a 3 T Tim TRIO or a 3 T VERIO MRI scanner (Siemens, Erlangen, Germany).

### Examination of impairments in sugar and fat perception

The twenty-five patients as well as the twenty-five healthy matched control participants were tested after written informed consent, around noon and without having eaten for five hours. Participants were comfortably seated in front of a table in an illuminated testing room. We tested sugar and fat perception with chocolate milkshakes that were produced by the Institute of Food Hygiene at the University of Leipzig (C.W. and P.B.). To test sugar perception, we offered three unlabeled probes of chocolate milkshake that were served in non-transparent cups. Each of the three milkshakes for the sugar tests (s1 to s3) consisted of 50 ml milk (3.5% fat content) and 5 ml Schwartau chocolate sauce (Schwartauer Werke, Bad Schwartau, Germany) consisting of glucose syrup, water, 7.5% cocoa powder, condensed milk, and carrageenan. For s2 we added 2.5 ml liquid sweetener (i.e., sodium cyclamate 12 g/100 ml, saccharin sodium 1.2 g/100 ml), and for s3 5 ml liquid sweetener. We did not apply any additional sweetener to s1. We used liquid sweetener instead of sugar since sugar added to milk changes its texture and viscosity. All three probes (50 ml) were placed in front of the participant on the table in random order. The participant was instructed to drink each probe and to rate its sweetness on a visual analog scale ranging from 0 (i.e., not sweet) to 10 (i.e., very sweet). To this end, participants were told to make a cross on a line (100 mm long) between 0 and 10. The distance in mm between 0 and the cross was used for further statistical analyses. Participants and controls were instructed to take small sips (not the whole 50 ml) and to swallow. After each probe, participants were instructed to rinse the mouth with 50 ml of non-sparkling mineral water and to eat a small piece of white bread after testing each probe. The three milkshakes for the fat tests (f1 to f3) consisted of different amounts of milk and cream. For f1 we used 50 ml milk (3.5% fat content) but no cream. For f2 we used 30 ml milk (3.5%) and 20 ml cream (32% fat content). For f3 we used 20 ml milk (3.5%) and 30 ml cream (32%). Like for the sugar tests, we added 5 ml Schwartau chocolate sauce to all three shakes (f1 to f3). The examination procedures were the same as described for the sugar test. The rating scale for fat stimuli ranged from “not creamy at all” to “very creamy”.

The step sizes between the three shakes of each category (sugar and fat) were first subjectively selected by two authors (T.W. and C.W.). Next, we performed pilot experiments in 6 healthy participants (3 men, mean age 36.3 +/− 7.8 years) to assess whether both steps (1–2 and 2–3) in both tests are of comparable sizes. In the main experiment, we asked whether patients experienced any differences in viscosity. For the sugar tests, no patient reported any differences in viscosity between s1, s2 or s3. For the fat tests, patients with an unaffected fat perception reported an increasing viscosity from f1 to f3.

The order of sugar and fat tests were counterbalanced across participants to exclude a systematic influence of progressive satiety on single probes.

We initially planned to repeat these tests three times and to also measure detection thresholds. The original protocol was 1 h 30 min long. Three out of the first five patients showed a drop in their attention already after 30 min. Two other patients cancelled the assessment after 45 min. For the following patients, we therefore excluded repetitions of taste procedures and detection thresholds which reduced the duration of the experiment to 20 min.

Individual ratings were mean corrected. We used a two-sample t-test for each of the six ratings (three for sugar, s1 to s3, and three for fat, f1 to f3) to assess differences between patients and controls. Significance threshold for the three sugar or fat ratings was Bonferroni-corrected with an adjusted p-value at 0.017. Analyses that proved significance were validated with two-sample permutation test using Welch’s t (B = 10000) as computed with R (https://www.r-project.org/). We additionally applied those permutation tests since there was much more variation in ratings within the patient group than the controls.

### Virtual lesion symptom mapping (VLSM)

We used MRIcroN (http://www.sph.sc.edu/comd/rorden/mricron) to manually delineate the lesion on every single transversal slice of the T1-weighted anatomical MRI images (Supplementary Figure 1). The two researchers (T.W. and B.P.) who mapped the lesions were blinded for the clinical deficits. With SPM12 (http://www.fil.ion.ucl.ac.uk/spm), we spatially normalized the T1-weighted anatomical images together with the lesion maps to the T1 MNI-template (i.e, Montreal Neurological Institute (MNI) standard space).

The normalized lesion maps together with the corresponding ratings were included into the voxel-by-voxel lesion-symptom mapping toolbox (VLSM 2.55: https://langneurosci.mc.vanderbilt.edu/resources.html) for Matlab R2017b (Natick, Massachusetts, USA, https://mathworks.com). To increase statistical power, patients with lesions in the right hemisphere were added after we flipped right/left orientation of normalized lesion masks. For each voxel, VLSM 2.55 divides patients into two groups according to whether they did or did not have a lesion affecting that voxel. Ratings were then compared for these two groups, yielding a t-statistic for each voxel^14^. The analysis was corrected for age, the individual lesion sizes and the time between MRI scanning and behavioral assessments (in days). In accordance with the publication that introduced VLSM 2.55^14^, significance threshold was Bonferroni-corrected with an adjusted p-value to the number of t-tests performed. Since two out of the six ratings showed significant differences between patients and controls (f1 and f3; see Results section for further details) we adjusted the p-value to 0.025. To assess differences between both brain hemispheres, we additionally analyzed the data of patients with left-sided and right-sided lesions separately. The raw (i.e., unthresholded) t-maps were visually compared to the result of the analysis containing the flipped lesion maps.

### Data availability statement

All data is available on request. The data will be anonymized. Please contact the corresponding author.

## Electronic supplementary material

Supplementary Information
