# Supplementary material for: Fat perception in the human frontal operculum, insular and somatosensory cortex
Source: Sci Rep. 2018 Aug 7;8:11825. doi: 10.1038/s41598-018-30366-0 (PMC6081453; doi:10.1038/s41598-018-30366-0)
Supplement: Supplementary file 1 — Supplementary Information [file 41598_2018_30366_MOESM1_ESM.pdf]

Supplementary material for

Fat perception in the human frontal operculum, insular and somatosensory cortex

Thomas Wistehube[1-3], Michael Rullmann[1-4], Claudia Wiacek[5,6], Peggy Braun[5,6], Burkhard Pleger[1-3,6-8]

[1]Department of Neurology, Max Planck Institute for Human Cognitive and Brain Sciences, Stephanstr. 1a, 04103 Leipzig, Germany

[2]Collaborative Research Centre 1052 Obesity Mechanisms, University of Leipzig, Leipzig, Germany

[3]IFB AdiposityDiseases, Leipzig University Medical Centre, Liebigstr. 20, 04103 Leipzig, Germany

[4]Department of Nuclear Medicine, University Hospital Leipzig, Liebigstr. 18, 04103 Leipzig, Germany

[5]Institute of Food Hygiene, Center of Veterinary Public Health, University of Leipzig, An den Tierkliniken 1, 04103 Leipzig, Germany

[6]BMBF nutriCARD, Center of Veterinary Public Health, University of Leipzig, An den Tierkliniken 1, 04103 Leipzig, Germany

[7]Department of Neurology, BG University Hospital Bergmannsheil, Ruhr-University Bochum, Bürkle-de-la-Camp Place 1, 44789 Bochum, Germany

[8]Collaborative Research Centre 874 Integration and Representation of Sensory Processes, Ruhr-University Bochum, Bochum, Germany

Correspondence should be addressed to: Burkhard Pleger, MD, Department of Neurology, BG University Clinic Bergmannsheil, Ruhr-University Bochum, Bürkle-de-la-Camp Platz 1, 44789 Bochum, Germany. Tel: 0049-163-9190215, Email: burkhard.v.pleger@rub.de

Supplementary Figure 1a

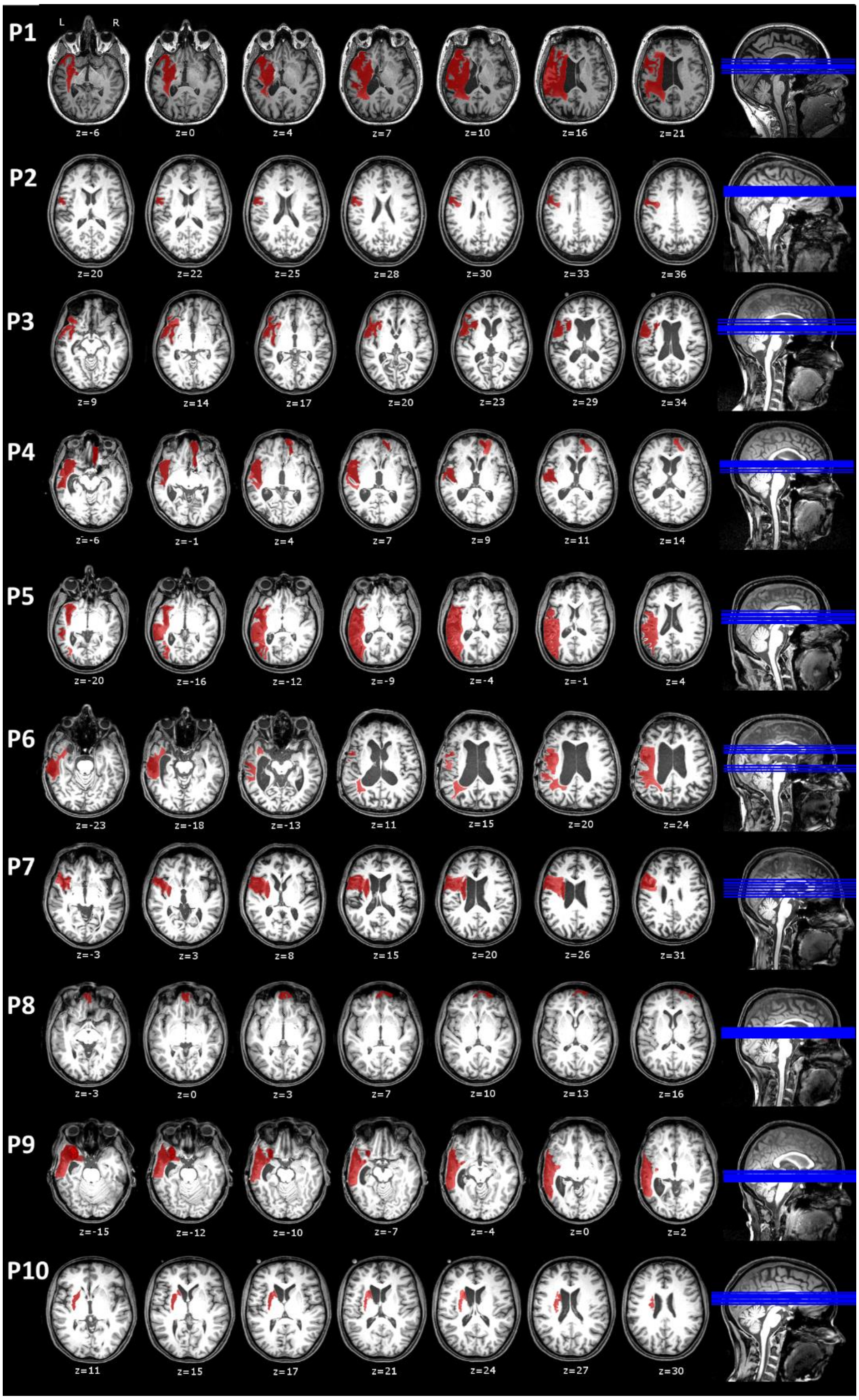

Supplementary Figure 1b

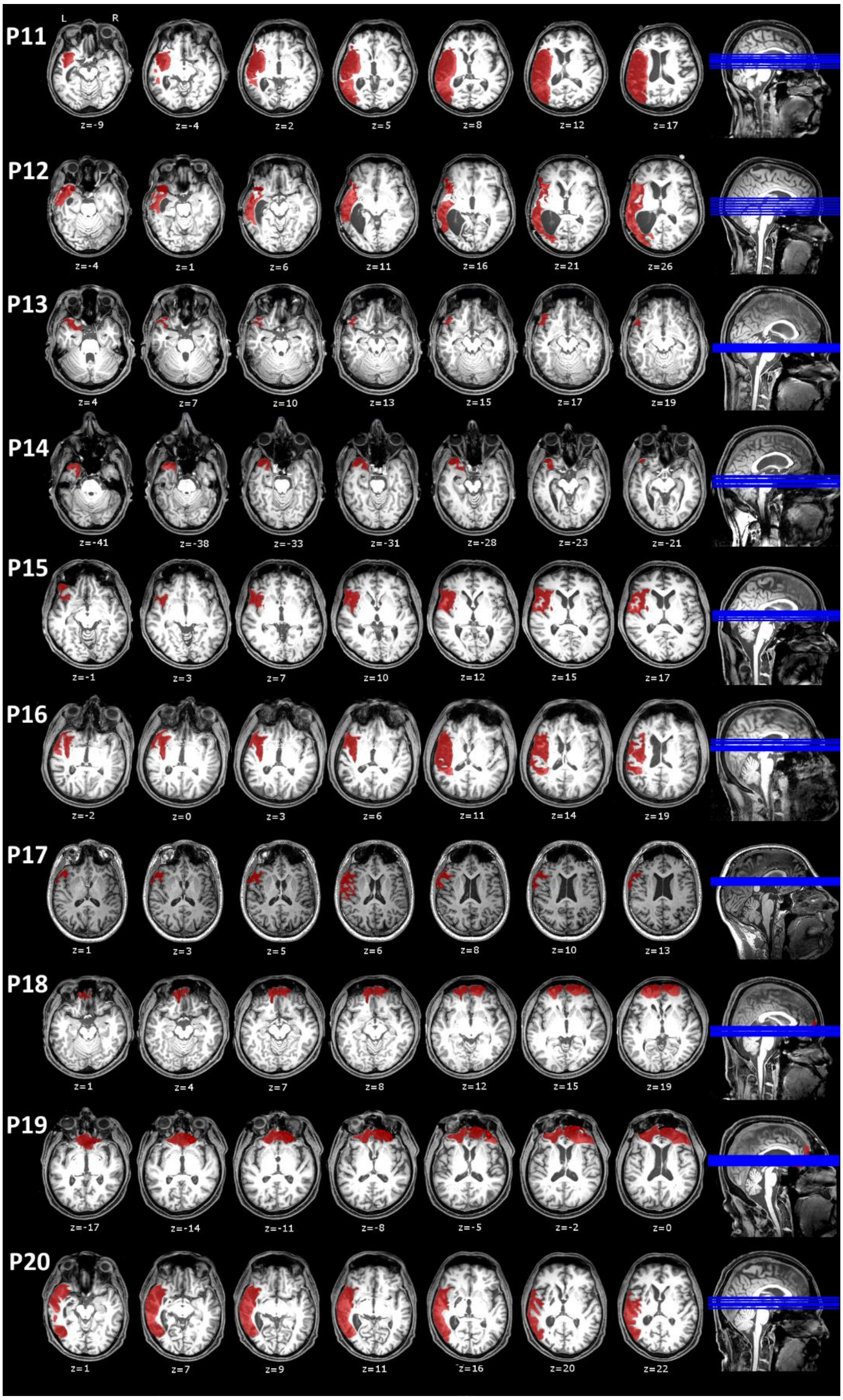

Supplementary Figure 1c

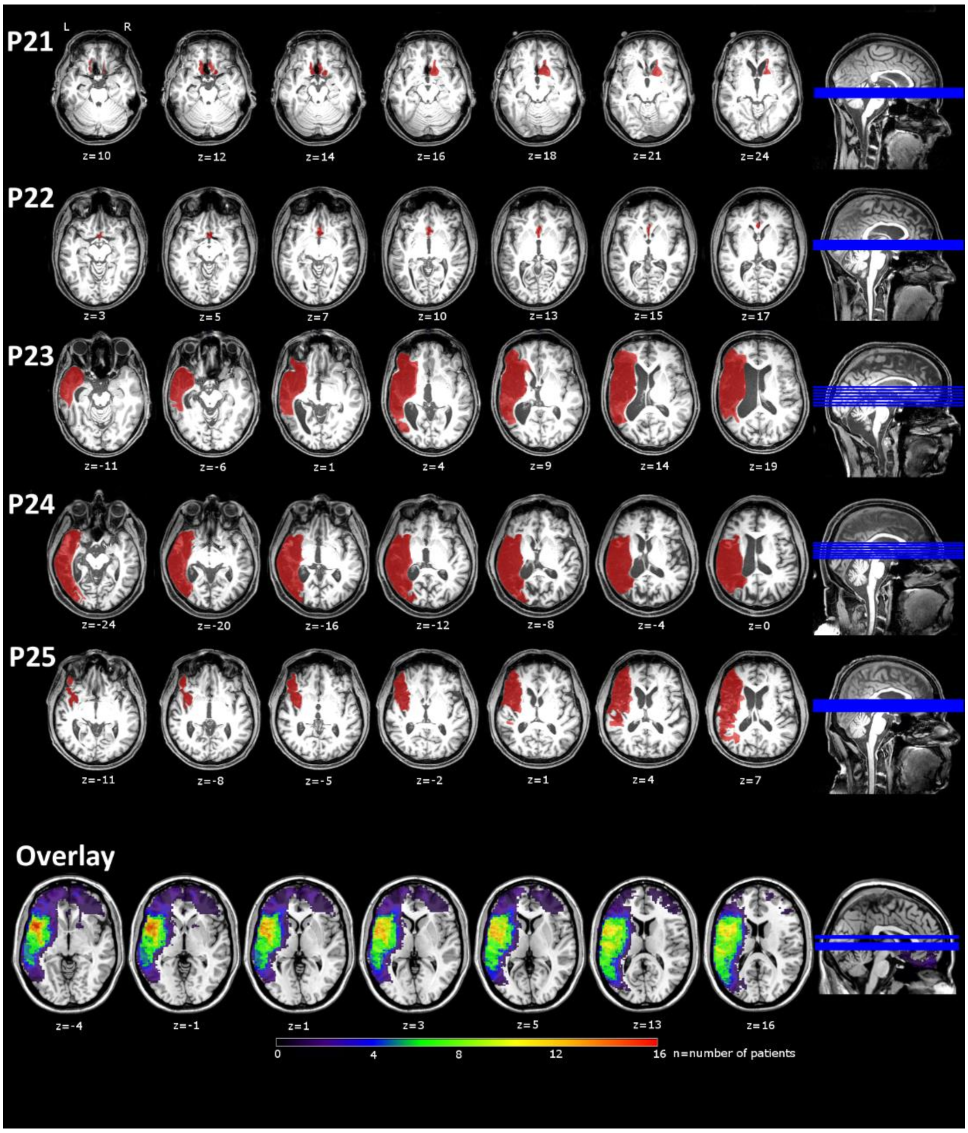

Supplementary Figure 1 a-c. Individual lesion pattern. Shown are the lesion pattern for each patient (a: P1-P10, b: P11-P20, c: P21-P25 plus overlay plot). The 'overlay' plot in Supplementary Figure 1c presents the overlay of all patients' lesion maps. The 'z' below each brain slice indicates the corresponding z-coordinate of that slice in MNI space. 'L' indicates the left and 'R' the right hemisphere. For the corresponding clinical data, please refer to Tab. 1 in the main text.

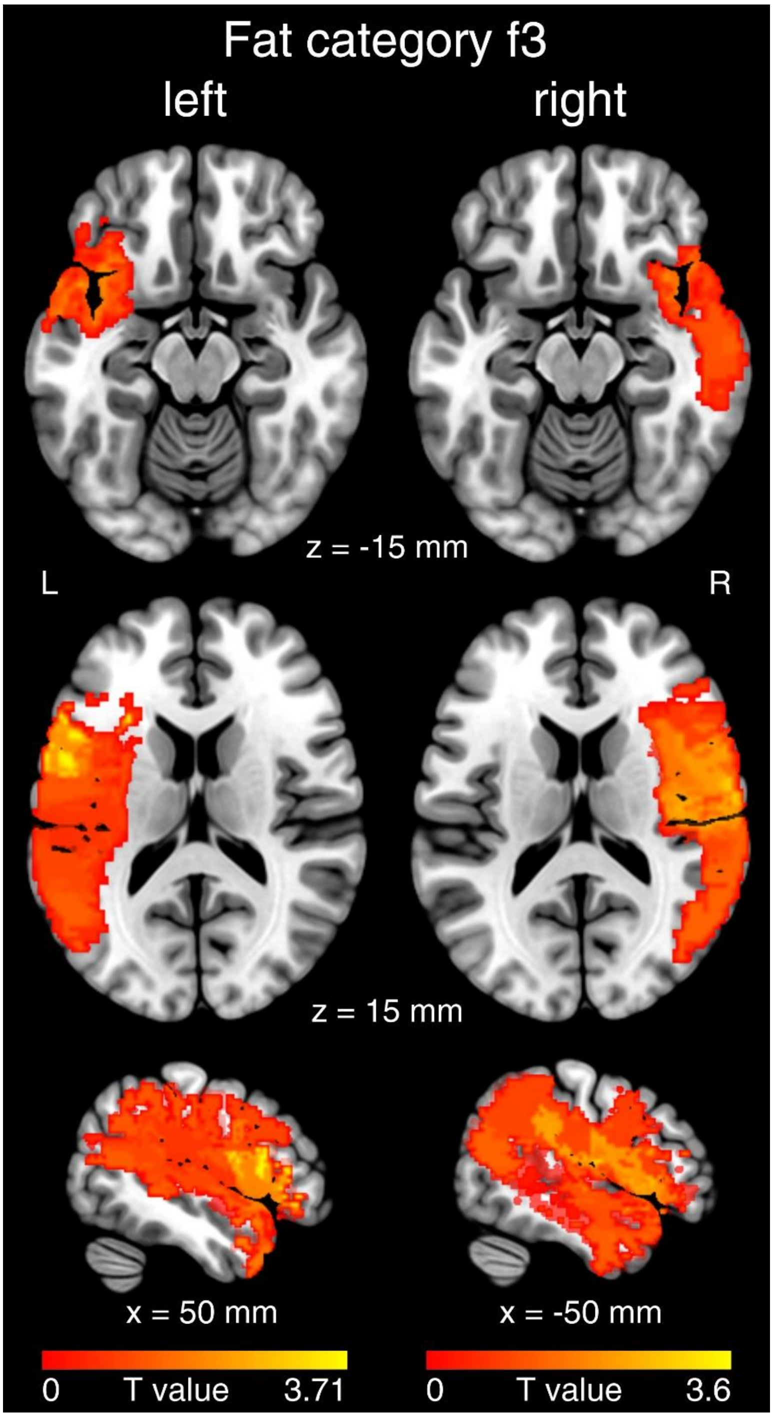

Supplementary Figure 2. Side-specific effects of deficits in fat perception (i.e. underrating f3). Separate analyses of patients with lesions in the left and right hemisphere who underrated f3. Unthresholded (i.e., raw) t maps of each hemisphere resembled the lesion pattern that we identified with the flipped analyses (see Figure 2) further supporting our interpretations. The ‘z’ and ‘x’ below brain slices indicates the corresponding z-coordinate or x-coordinate of that slice in MNI space. ‘L’ indicates the left and ‘R’ the right hemisphere.

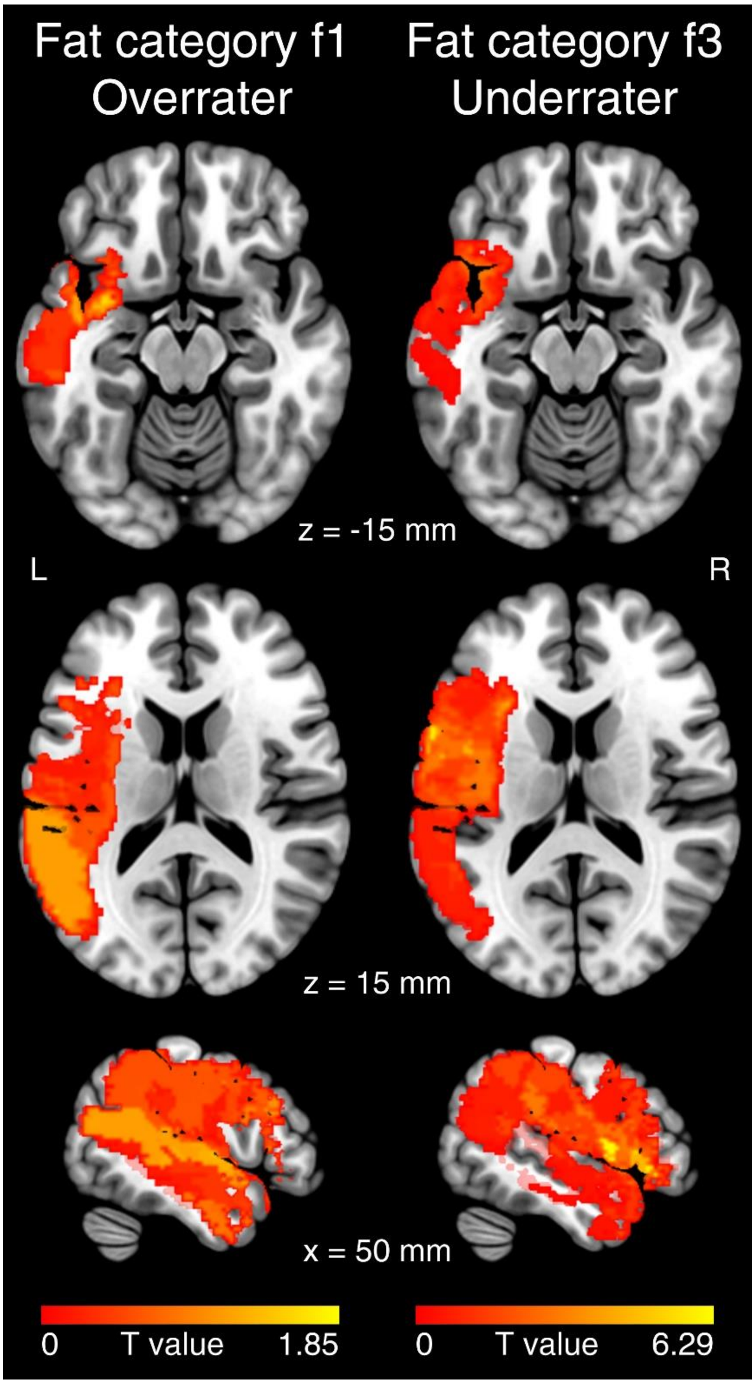

Supplementary Figure 3. Overlay of lesions of patients who overrated f1 and of those who underrated f3. The comparison must be handled carefully since we could not compare lesion maps statistically. Visual comparison suggests that patients who underrated f3 presented lesions in the frontal operculum, whereas patients who overrated f1 did not show lesions in this region. The ‘z’ and ‘x’ below brain slices indicates the corresponding z-coordinate or x-coordinate of that slice in MNI space. ‘L’ indicates the left and ‘R’ the right hemisphere.
